# Active Biopolymeric Packaging for Fatty Foods: Chitosan/Electrospun Zein Bilayer Membranes with Natural Additive

**DOI:** 10.1021/acsomega.5c11399

**Published:** 2026-06-09

**Authors:** Luisa Bataglin Avila, Paula da Cruz Pedroso, Caroline Costa Moraes, Guilherme Luiz Dotto, Gabriela Silveira da Rosa

**Affiliations:** † Chemical Engineering, 186060 Federal University of Pampa (UNIPAMPA), Maria Anunciação Gomes Godoy Avenue, Bagé, Rio Grande do Sul 96413-172, Brazil; ‡ Graduate Program in Materials Science and Engineering, Federal University of Pampa(UNIPAMPA), Maria Anunciação Gomes Godoy Avenue, Bagé, Rio Grande do Sul 96413-172, Brazil; § Research Group on Adsorptive and Catalytic Process Engineering (ENGEPAC), Federal University of Santa Maria, Roraima Avenue, Santa Maria, Rio Grande do Sul 97105-900, Brazil

## Abstract

This study aimed to develop zein-chitosan bilayer membranes incorporated with lyophilized jaboticaba epicarp extract (LJEE) for applications in food packaging. The LJEE was initially characterized for its total phenolic content (TPC), antioxidant activity (AA), total anthocyanin (TA), and phenolic profile via HPLC analysis. The characterization revealed promising properties, with TPC, AA, and TA values of 89.33 ± 3.46 mgGAE g^–1^, 70.85 ± 1.85%, and 416.74 ± 9.43 mg cn-3-glu/100 g, respectively. Notable bioactive compounds identified included gallic acid, caffeic acid, *p*-coumaric acid, trans-ferulic acid, kaempferol, and cyanidin-3-glucoside (cn-3-glu), present in higher concentrations than other compounds. The bilayer membranes were produced using chitosan film (first layer) and electrospun zein fiber (second layer). LJEE was incorporated into the zein solution, producing an active bilayer membrane (ABM). A control bilayer membrane (CBM), without LJEE, was also formulated. Zein solutions were evaluated according to their surface tension and contact angle with the chitosan film surface, and the addition of LJEE improved adhesion. The incorporation of LJEE into the membranes significantly reduced water solubility and water vapor permeability. The mechanical properties of the bilayer membranes were favorable, particularly in terms of elongation at break. Morphological analysis of the electrospun fibers showed homogeneous fibers with average diameters of 975 and 675 nm for CBM and ABM, respectively. FTIR analysis demonstrated interactions between the components of the extract and the biopolymers, while thermal analysis demonstrated that the addition of the extract did not negatively affect membrane stability, indicating that the bilayer configuration provides greater structural stability than the chitosan film alone. The migration of bioactive compounds from ABM to different food simulants was also investigated, and better results were observed in oil. Additionally, ABM exhibited antimicrobial activity in the oil simulant, effectively inhibiting *S. aureus* and *E. coli*. The results suggest that these bilayer membranes with LJEE can be used as a promising material for active food packaging, preferably serving fatty food products.

## Graphical abstract

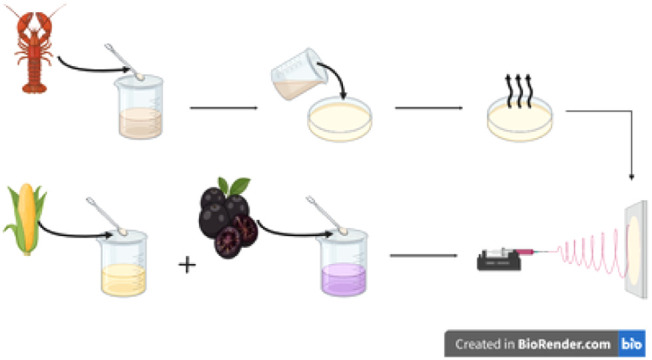

## 1 Introduction

Nowadays, petroleum-based plastics are commonly used in food-packaging materials, mainly due to their low price and good mechanical and barrier properties. However, a lot of attention has been paid to their widespread use due to their dangerous environmental effects and associated health risks to consumers. Concurrently, the trend toward healthier lifestyles has boosted the search for natural additives to be used in food packaging, mainly due to the adverse effects caused by synthetic ones.^1–5^

Efforts have been made to develop commercially viable materials based on biopolymers and natural additives for use in food packaging.^6–9^ More recently, to improve mechanical and barrier properties, new approaches have been reported, with emphasis on multilayer systems and electrospinning technologies. The first technology is characterized by a set of layers that perform different functions and the possibility of combining different biopolymers and techniques for their formation. Therefore, the main advantage of a multilayer system is related to the inclusion of the properties resulting from each biopolymer used, as well as the techniques used, in a single packaging structure.^10,11^ On the other hand, electrospinning is a promising technique for producing fibrous materials with a high surface-to-mass ratio, porosity, and encapsulation efficiency of bioactive compounds. These properties are mainly related to submicrometer- or nanometer-scale fibers, attracting attention and interest from the food packaging industry due to the possibility of controlled release of bioactive compounds.^12,13^

In this sense, among the wide variety of biopolymer resources available in nature, chitosan and zein stand out because of their properties and abundance. Chitosan is a polysaccharide obtained mainly from residues of crustaceans and is considered the second most abundant polymer after cellulose, while zein is the main protein in corn grain but is not used in the human diet due to its low nutritional value.^14–17^ Several studies in the literature report on the use of these biopolymers for food packaging applications.

The use of natural resources of active compounds is a growing trend in the food packaging sector.^18^ In this scenario, jaboticaba plays an important role as a rich source of bioactive compounds. Commonly recognized for its flavor, mainly associated with the pulp, jaboticaba has attracted attention due to its high levels of anthocyanins, which exhibit important properties such as antimicrobial and antioxidant activities. These compounds are especially concentrated in the epicarp, which presents a characteristic dark color. The antimicrobial and antioxidant activity of jaboticaba epicarp makes it promising for application as a natural additive in food packaging and can be incorporated into the matrix of different biopolymers.^6,10,19–23^

Although natural extracts present interesting characteristics from the point of view of bioactive compounds, their effectiveness when incorporated into a biopolymeric matrix and their release capacity when in contact with a food matrix require specific studies. In this framework, the present study focused on the development of a bilayer membrane based on chitosan and zein, incorporated with lyophilized jaboticaba epicarp extract (LJEE), designed for active packaging applications. In this configuration, chitosan forms a robust bottom layer, while zein fibers function as the active compartment for extract incorporation and controlled release. There is still limited work on chitosan/zein bilayer systems for fatty foods, especially those leveraging hydrophobic phases to enhance phenolic release and antimicrobial effectiveness. The bilayer was evaluated based on physicochemical characterization, antioxidant and phenolic release, and antimicrobial activity in food simulants.

## 2 Material and Methods

### 2.1 Reagents

For biopolymeric membrane preparation, chitosan (Êxodo Científica, Sumaré, SP, Brazil), glycerol (Alphatec, WF, Pelotas, RS, Brazil), zein (Sigma-Aldrich, St. Louis, MO, USA), and acetic acid (Synth, Diadema, SP, Brazil) of analytical grade were used. For bioactive compound analysis, all reagents were purchased from Sigma-Aldrich (St. Louis, MO, USA), including 2,2-diphenyl-1-picrylhydrazyl (DPPH), Folin-Ciocalteu’s phenol, and anhydrous sodium carbonate. For the antimicrobial test, Nutrient broth and Müller-Hinton broth (Himedia, WF, Pelotas, RS, Brazil) were used. The bacterial strains used in the antimicrobial test were *Escherichia coli* (11303–10G) and *Staphylococcus aureus* (WDCM 00032), obtained from Sigma–Aldrich (Sigma–Aldrich Brasil Ltd).

### 2.2 Production and Characterization of Lyophilized Jaboticaba Epicarp Extract (LJEE)

Jaboticaba fruits (*Plinia cauliflora*) were collected from a private farm in Santa Maria (−29.88926, −53.87125), Rio Grande do Sul, Brazil. The methodology adopted for sample preparation involved pulping, sanitizing, drying, and grinding under conditions previously established by Avilaet al.^10^

The jaboticaba epicarp extract (JEE) was produced by the maceration technique in a Dubnoff metabolic bath (SOLABSL-157/30, Piracicaba, Brazil), using a particle-to-solvent ratio (aqueous ethanol −40%) of 1:100 for 2 h at 80 °C, as described by Filho et al.^24^ with some modifications. After the extraction stage, the samples were vacuum-filtered, rotary-evaporated, frozen, and freeze-dried to obtain LJEE.

The LJEE produced was resuspended in 70% ethanol at a proportion of 3% (w/v), the same concentration that will be used in the formulation of the final product. The resuspended LJEE was evaluated according to total phenolic content (TP), using the spectrophotometric method adapted from the one established by Singleton & Rossi (1965)^25^ and using a standard curve with different concentrations (72–1800 mg L^–1^) of gallic acid. The antioxidant activity (AA) of the extracts was also analyzed by Brand-Williams et al.^26^ method. The analysis of total anthocyanins (TA) was carried out according to the method described by Sripakdee et al.^27^ with some modifications, in which the results are measured based on a standard calibration curve of Cyanidin-3-Glucoside (Cn-3-Glu) at concentrations ranging from 5–100 mg L^‑1^.

The quantitative analysis of phenolic compounds was performed using a High-Performance Liquid Chromatography (HPLC) system, Agilent 1260 Infinity (Agilent Technologies, Santa Clara, CA, USA), equipped with a quaternary pump (Series 1200) and a diode-array detector (DAD, Series 1260). Separation was conducted on a reverse-phase LC Eclipse Plus C18 column (150 × 4.6 mm, 5 μm, Supelco, Bellefonte, PA, USA), maintained at 30 °C.

The mobile phase consisted of solvent A (0.2% acetic acid), solvent B (methanol), and solvent C (acetonitrile). A gradient program A/B/C was applied as follows: 0 min—96/2/2%; 5 min—80/10/10%; 10 min—70/15/15%; 20 min—50/25/25%; and reconditioning at 96/2/2% from 30 to 40 min. The flow rate and injection volume were 1.0 mL·min^–1^ and 20 μL, respectively, as presented in ref. 24.

Lyophilized JEE samples were dissolved in 40% ethanol (v/v), filtered through a 0.45 μm PVDF membrane, and analyzed in duplicate. Detection was performed at 280 nm (phenolic acids) and 520 nm (anthocyanins). Phenolic compounds were identified by comparison of retention times with Sigma-Aldrich standards (gallic acid, caffeic acid, *p*-coumaric acid, trans-ferulic acid, kaempferol, and cyanidin-3-glucoside) and quantified using standard calibration curves. Results were expressed in mg·100 g^–1^ of dry matter.

### 2.3 Bilayer Membranes Production and Characterization

The production and characterization of bilayer membranes are summarized in Figure 1.

**Figure 1:**
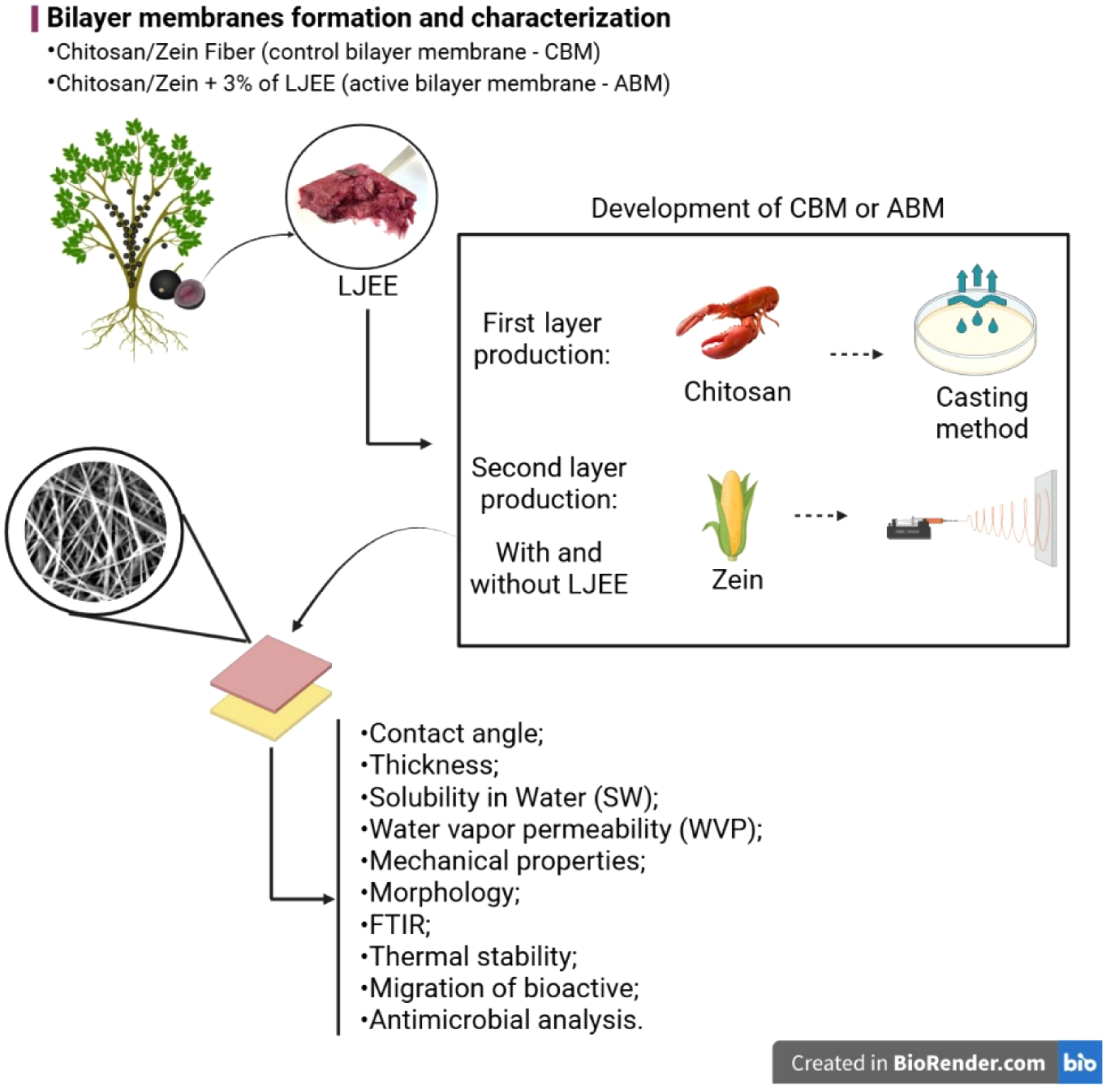
Production and characterization of bilayer membranes. Image created in BioRender.com

The bilayer membranes were produced through a combination of casting and electrospinning techniques. The design of the layer structure included both inner and outer layers. The inner layer, consisting of a thin chitosan film, was obtained by the casting method following the proportions and conditions described by Soares et al.^28^ Thus, 1 g of biopolymer, 75 mL of 1% (v/v) acetic acid solution, and 0.3 g of plasticizer (glycerol) were used. The film-forming solution was first allowed to stand overnight and was homogenized with the plasticizer in a mechanical shaker under light agitation for 10 min. Finally, the samples were poured into polystyrene Petri dishes (150 mm diameter) and dried in a convective dryer at 40 °C for 24 h. The thin films were removed from the plates and stored in an environment at 50% relative humidity and room temperature.

The outer layer, consisting of zein electrospun fibers, was obtained by the electrospinning technique following the methodology described by Antunes et al.^29^ First, the zein solution was prepared using 0.9 g of biopolymer and 3 mL of 70% (v/v) ethanol, which was stirred at room temperature for 1 h. A zein solution with LJEE was obtained using the same method, however, the incorporation of LJEE into the biopolymeric matrix was carried out by previously dissolving 3% (w/v) of LJEE in 70% (v/v) ethanol for subsequent addition of zein.^19^ After that, the solutions produced were electrospun onto the thin chitosan film, which was placed on a metal collector. The flow rate of the polymer solution was fixed at 1 mL h^–1^ and a voltage of 18 kV was used. The distance between the collector and the needle was maintained at a constant 10 cm for all samples, with the process conducted in an environment where temperature and relative humidity were controlled at 23 ± 2 °C and 45 ± 2%, respectively. The developed bilayer membranes were called the control bilayer membrane (CBM) and the active bilayer membrane (ABM).

The surface tension and contact angle of the samples were evaluated at room temperature through the pendant-drop and sessile-drop methods, respectively. A drop of zein solution (20 μL), with and without LJEE, was deposited over the surface of the chitosan thin film, and the properties were analyzed using an optical tensiometer (One Attention – Theta Instrument) equipped with a camera. For each formulation, a single batch-prepared solution was used, from which eight independent drops were dispensed. The results were expressed as the mean ± standard deviation of these technical replicates (n), randomly distributed over the biopolymeric surface.

The thickness of bilayer films was determined using a digital micrometer (Insize-IP65, São Paulo, Brazil) with an accuracy of 0.001 mm. The solubility in water (SW) was investigated according to the methodology described by Gontard et al.^30^ and the water vapor permeability (WVP) was performed following the standard.^31^ The mechanical properties of the bilayer membranes were analyzed by measuring the tensile strength (TS) and elongation at break (E) according to ASTM Standard D882–09,^32^ using a texture analyzer (STABLE MICRO SYSTEM—TA.XT.plus, Surrey, UK).

The surface morphology analyses were carried out using the scanning electron microscope (SEM) (Jeol, JSM-6060LVAkishima, Tokyo, Japan). The identification of the functional groups in the samples, as well as the interaction between the extract and biopolymer, was investigated using Fourier transform infrared (FTIR) analyses in a spectrometer (Shimadzu, Prestige 21, Nakagyo-ku, Kyoto, Japan). The thermal stability was performed using a thermogravimetric instrument (Shimadzu, TGA 50, Kyoto, Japan), with conditions described by Avila et al.^19^

The active potential of bilayer membranes was determined by the migration of bioactive compounds and antimicrobial analysis. First, the migration test was carried out in a food matrix using the method described by RDC no. 51.^33^ For this purpose, four food simulants were used: distilled water, aqueous acetic acid solution 3% (v/v), ethanol 10% (v/v), and oil. Samples were prepared: 0.3 g of bilayer membranes were immersed in 15 mL of each type of food simulant, vortexed for 2 min, sonicated for 5 min, and kept at rest for 4 h at 25 °C. The supernatant was evaluated according to antioxidant activity and total phenolic content using the methodologies described in Section 2.4.

The food simulant that provided the best results for antioxidant activity and total phenolic content was used in the antimicrobial analysis. The samples were prepared according to the same methodology used in the migration test. After the migration time, supernatant aliquots were evaluated following the microdilution method of the Clinical and Laboratory Standards Institute,^34^ and the growth inhibition of Gram-positive microorganisms, *Staphylococcus aureus* (*S. aureus*) and Gram-negative microorganisms, *Escherichia coli* (*E. coli*) was evaluated. The analysis was carried out in a microplate spectrophotometer (KASUAKI, DR-200BS-NM-BI, Wuxi, China). Afterward, the microplate was incubated at 37 °C for 16 h. A control (inoculum without sample) was also included in the microplate. The contents of the wells were mixed before reading the absorbances at 620 nm, and the results were expressed as percentages.

### 2.4 Statistical Analysis

Experimental data were statistically analyzed by Statistica software, version 10.0 (SAS Institute, Cary, NC, USA). Pairwise comparisons of means were performed using the *t*-test. For multiple comparisons, analysis of variance (ANOVA), followed by Tukey’s post hoc test, was applied. All tests were performed with a significance level of 95% (*p* < 0.05).

## 3 Results and Discussion

### 3.1 Evaluation of the Bioactive Content of the Extract

The characterization of LJEE showed promising results regarding total phenolic content, antioxidant activity, and total anthocyanin, which were 89.33 ± 3.46 mg_GAE_ g^–1^, 70.85 ± 1.85%, and 416.74 ± 9.43 mg cn-3-glu/100 g, respectively. These values fall within the range commonly reported in the literature; however, differences across studies may be explained by variations in preprocessing and extraction approaches. The results found are similar to those reported in the literature,^24^ which reported a content of total phenolic compounds for jaboticaba epicarp extract of 122.63 ± 1.79 mg_GAE_ g^–1^ in their study on extraction conditions and the drying process of the raw material. In this case, the authors^24^ used a combination of convective drying and freeze-drying followed by ethanolic maceration (40%) at 88 °C, conditions that typically enhance phenolic diffusion and recovery. In another study,^19^ a value closely approximating the results presented in this study was reported (98.88 ± 1.79 mg_GAE_ g^–1^), also obtained from the maceration technique at 88 °C for 2 h and using ethanol 40% as the solvent.^35^ They also evaluated total phenolic recovery using hydroethanolic extraction under milder temperature conditions (57 °C), reporting 106 mg GAE g^–1^, illustrating the sensitivity of extraction yield to solvent concentration and temperature. Overall, such variability among authors can be largely attributed to differences in drying (convective vs freeze-drying), solvent polarity, extraction temperature, time, and solid–liquid ratio, all factors known to influence the efficiency of phenolic recovery from plant biomass.

Antioxidant activity is an important property that has received significant attention, mainly due to the possibility of mitigating oxidative processes, which form free radicals and peroxides, leading to nutritional losses in food.^36^ In this sense, the high result of the antioxidant activity of LJEE is in accordance with reports in the literature and corroborates the result of total phenolic content, since, according to Barros et al.,^37^ the antioxidant potential is one of the main characteristics of these compounds.^19^ A study reported a value of 81.00 ± 0.72% for JEE, using an ethanolic solvent (40%) with a maceration technique during 2 h at 88 °C, while NAN et al.^38^ found a value of 86.31% using a 1:2 water:ethanol solution as the solvent and the technique of maceration followed by percolation, during 96 h.

These variations among studies^19–37^ can be explained by differences in solvent composition, extraction duration, and processing intensity, since more polar solvents and longer extraction times typically enhance the recovery of antioxidant constituents. Additionally, mild degradation of thermolabile compounds may occur depending on the temperature and agitation, also influencing measured antioxidant activity.

Similar to total phenolic content, total anthocyanins also play important roles, particularly in antioxidant and antimicrobial activities. According to Sanhueza et al.,^39^ these compounds do not act independently but rather synergistically. Therefore, the results presented here are in accordance with those proposed in the literature. Lenquiste et al.^40^ evaluated the effect of different extraction solvents on the recovery of bioactive compounds from jaboticaba epicarp and found values of 630.46 ± 21.76 and 404.56 ± 35.85 mg cn-3-glu/100 g for the methanolic and aqueous extracts, respectively. Romualdo et al.^41^ showed a value of 802.89 ± 22.88 mg cn-3-glu/100 g for total anthocyanins present in JEE obtained from ultrasound extraction in ultrasonic bath using a methanol/formic acid solution (90:10 v/v).

The phenolic profile of LJEE was also evaluated, and the results are shown in Table 1.

**Table 1:** Identified Phenolic Compounds in Lyophilized JEE^a^

Compounds	Concentration (mg 100 g^–1^)
Gallic acid	31.33 ± 1.61
Caffeic acid	20.14 ± 0.40
*p*-Coumaric acid	45.74 ± 1.45
Trans-Ferulic acid	26.50 ± 0.76
Kaempferol	50.26 ± 1.70
Cyanidin-3-glucoside	935.45 ± 16.70

a Mean ± standard deviation (*n* = 2).

Cn-3-Glu is clearly the main compound in LJEE, corroborating what is described in the literature.^10,42^ Moreover, the JEE presented other important compounds, although in concentrations lower than cyanidin-3-glucoside, such as gallic acid, caffeic acid, *p*-coumaric acid, trans-ferulic acid and kaempferol. Romualdo et al.^41^ reported similar cyanidin-3-glucoside content for *Myrciaria jaboticaba* peel, 789.47 ± 20.95. Pimenta Inada et al.^43^ reported values ranging from 346 ± 8 to 818 ± 28 mg/100 g for cyanidin-3-glucoside from unprocessed jaboticaba peels and seeds, and processed with high hydrostatic pressure (200, 350, and 500 MPa). da Silva Jyp et al.^44^ found 798.26 ± 26.71 mg/100 g for cyanidin-3-glucoside in lyophilized jaboticaba epicarp and also reported 29.90 ± 0.76 mg/100 g of gallic acid content. Avila et al.^19^ and Filho et al.^24^ reinforce that even though other compounds are present in concentrations lower than the main anthocyanin, they play an important role related to its antioxidant and antimicrobial actions. It is also important to highlight that combinations of bioactive compounds improve the benefits of the extract compared to the use of individual compounds, an effect known as a synergistic effect.^45–49^

Differences between anthocyanin yields reported in the literature are strongly influenced by solvent polarity, temperature, and the use of enabling technologies (such as ultrasound or high hydrostatic pressure) capable of disrupting cell walls and improving pigment release. Furthermore, anthocyanins are highly sensitive to pH and thermal exposure, which explains the wide numerical ranges observed across studies.

It is possible to infer that the extraction procedures allowed the recovery of bioactive compounds from the jabuticaba epicarp, as evidenced by the high levels of phenolic compounds, anthocyanins, and antioxidant activity in the LJEE. This fact notably demonstrates the promising use of an agro-industrial residue as a raw material for the development of a natural additive.

Given the diversity of extraction approaches reported in the literature, varying in solvent systems, temperature, time, and pretreatment, such variability reinforces the importance of optimizing conditions tailored to each raw material. The results obtained in this work demonstrate that freeze-drying combined with ethanolic extraction is effective for preserving and recovering compounds of technological relevance for active packaging applications.

### 3.2 Bilayer Membranes Characterization

To better understand the fiber formation process, as well as the deposition of the zein layer on the chitosan layer, the surface tension and contact angle of both biopolymeric formulations were investigated. The results are presented in Figure 2.

**Figure 2:**
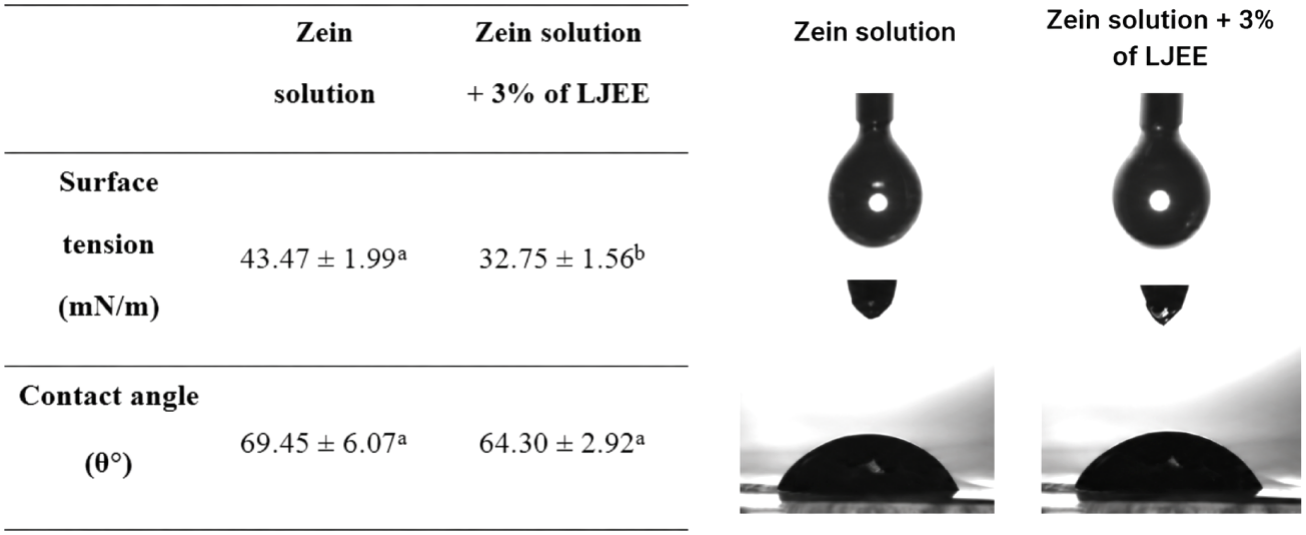
Surface tension and contact angle of zein solution and zein solution with LJEE. Mean ± standard deviation (*n* = 8). Different letters in the same line indicate significant differences between the samples for the *t*-test (*p* < 0.05).

As shown in Figure 2, the contact angle is mainly governed by the interfacial interactions between zein and chitosan. While zein exhibits hydrophobic characteristics, chitosan provides polar functional groups, resulting in a balance that determines the overall wettability. Thus, the observed behavior reflects the combined physicochemical properties of both polymers, being primarily governed by polymer–polymer interactions rather than contributions from phenolic groups in the extract. These results reflect high wettability and good adhesion between the layers.^58^

Regarding the surface tension of the zein solutions, it was possible to observe a reduction by the addition of LJEE. This behavior can be attributed to the presence of phenolic compounds in LJEE, which contain both hydrophilic groups (such as hydroxyl groups) and hydrophobic aromatic structures. This amphiphilic nature allows these molecules to migrate to the liquid–air interface, reducing cohesive forces within the solution and, consequently, lowering surface tension.^50^ Similar results are reported by Yang et al.,^59^ who evaluated the surface tension of zein solutions with different concentrations of sorghum extract and observed a decrease in this parameter by the addition of extract, with values from 47.5 ± 2.1 mN/m for pure zein to 45.1 ± 2.5 mN/m for the zein solution with the highest extract concentration (20 wt %). According to Abutaleb et al.,^60^ surface tension plays a crucial role in the formation of electrospun fibers.

Bilayer membranes were characterized, and the results are presented in Table 2.

**Table 2:** Bilayer Membranes Characterization[Table-fn tbl2fn1]

	CBM	ABM
Thickness (mm)	0.36 ± 0.07^b^	0.95 ± 0.07^a^
Solubility in Water (%)	19.45 ± 0.30^a^	8.19 ± 0.19^b^
Water Vapor Permeability (g·m^–1^·s ^–1^·Pa^–1^)	4.52 x 10^–10^ ± 5.14 x 10^–12a^	1.66 x 10^–10^ ± 5.14 x 10^–12b^
Tensile strength (MPa)	0.65 ± 0.13^a^	2.75 ± 0.02^b^
Elongation at break (%)	35.82 ± 2.76^a^	25.96 ± 3.28^a^

i Mean ± standard deviation (*n* = 3). Different letters in the same line indicate significant differences between the samples for the *t*-test (*p* < 0.05).

The results presented in Table 2 suggest that the addition of LJEE into the zein matrix promoted a significant increase in membrane thickness. The addition of LJEE, rich in phenolic compounds, can increase the solid content of the system and promote intermolecular interactions, especially hydrogen bonds, between the hydroxyl groups of the phenolics and the polymer chains. Liao et al.^51^ report that these interactions directly influence the structural organization of the polymer matrix, potentially resulting in modifications to the density and architecture of the material.

Furthermore, the increase in solid content in the film-forming solution is directly related to the increase in membrane thickness, as already reported for systems containing phenolic extracts.^52^ The incorporation of compounds capable of establishing hydrogen bonds can also favor the retention and deposition of material in the matrix, contributing to an increase in the final thickness. Figueroa-Lopez et al.^61^ reported a small increase in the thickness of the multilayer system based on polycaprolactone fibers and gelatin film by adding black pepper oleoresin into the fiber layer.Martins et al.^62^ also reported similar behavior when developing cellulose-based bilayer films with cinnamaldehyde and using solvent casting and electrospinning techniques. Zhang et al.^63^ reported values for chitosan/bilayer films produced by the casting method, ranging from 0.140 to 0.151 mm for different proportions of biopolymers.

The solubility in water also showed a significant difference between samples, and the addition of JEE decreased this parameter. This behavior can be attributed to the presence of phenolic compounds in the extract, which are capable of establishing intermolecular interactions, particularly hydrogen bonding, with the functional groups of the polymeric matrix.^53^ These interactions reduce the availability of free hydrophilic sites, limiting the interaction between the polymer chains and water molecules.^54^

In addition, the incorporation of LJEE may promote the formation of a more cohesive and compact network structure, restricting the penetration and diffusion of water into the material. The partial hydrophobic character of phenolic compounds, due to their aromatic rings, may also contribute to decreasing the overall affinity of the membrane for water.^53^ As a result, the combined effect of reduced availability of polar groups and increased structural integrity leads to lower solubility.^55^ The same behavior was reported by Kanatt et al.^64^ for PVA-Gelatin film added with Amaranthus leaf extract and observed a reduction by the addition of the extract. The authors found values of 53.10 ± 0.58% and 88.13 ± 1.22% for membranes with and without extract, respectively, and related this fact to the number of free hydroxyl groups from the extract that can form hydrogen bonds between the polymers. Zhao et al.^65^ developed chitosan/zein films with different concentrations of *Rosa roxburghii* Tratt leaves extracts and reported higher values in the range of 31.66 ± 1.24 to 25.81 ± 0.94%. According to Puscaselu et al.,^66^ solubility in water is an important parameter to be evaluated when developing a food packaging material, as it provides substantial information for choosing the material to be packaged for products with high/low moisture content.

Regarding water vapor permeability, it is possible to observe that the addition of LJEE into the biopolymeric matrix resulted in a significant reduction in this parameter. Likewise, Xu et al.^67^ reported that chitosan/zein films containing essential oil nanoemulsions and nanoparticles reached values ranging from 2.41 × 10^–9^ to 2.48 × 10^–9^ g·m^–1·^s^–1^·Pa^–1^ and from 2.60 × 10^–9^ to 3.03 × 10^–9^ g·m^–1^·s^–1^·Pa^–1^, respectively, depending on EO concentration.

The positive effect of incorporating LJEE can be attributed not only to the increase in bilayer membrane thickness, which hinders vapor diffusion, but also to molecular interactions between the extract and the biopolymeric matrix. Phenolic compounds present in LJEE display an amphiphilic character, containing both hydrophilic hydroxyl groups and hydrophobic aromatic rings.^67^ When incorporated into the zein–chitosan network, these molecules may establish hydrogen bonding with available polar groups, reducing their accessibility to water molecules while simultaneously increasing the hydrophobic character of the internal phase of the film.^68^ In addition, their integration into the biopolymeric network can promote a more compact structure and create a more tortuous path for moisture transport. Consequently, these combined effects decrease the affinity of the material for water and restrict vapor migration across the structure, which explains the enhanced barrier behavior observed. According to Li et al.,^68^ adequate water vapor barrier properties are desirable in food packaging materials, as they contribute to extending product shelf life by limiting moisture transfer between the food and the surrounding environment.

The mechanical properties were also investigated regarding tensile strength and elongation at break. It is possible to observe in Table 2 that the tensile strength results showed a significant improvement due to the addition of the extract, while the elongation at break was not affected. This behavior can be attributed to the presence of phenolic compounds in the extract, which are capable of forming intermolecular interactions, particularly hydrogen bonds, with the polymeric chains.^53^ These interactions enhance interchain cohesion and restrict the mobility of polymer segments, resulting in improved resistance to applied stress.^56^

At the same time, the absence of significant changes in elongation at break suggests that the flexibility of the system was preserved.^57^ This may indicate that, although additional interactions were established, they were not sufficient to severely hinder chain mobility or induce excessive rigidity in the network.^56^ The balance between reinforcement and flexibility is likely related to the concentration of the extract and the distribution of intermolecular interactions within the matrix.^53^ Furthermore, the results of both parameters are similar to those proposed in the literature. Figueira et al.^69^ developed a polycaprolactone-hyaluronic acid/chitosan-zein electrospun bilayer membrane and found values of 2.17 ± 0.62 MPa and 23.09 ± 3.07% for tensile strength and elongation at break, respectively. Ullah, et al.^70^ also investigated the tensile strength of electrospun zein-polycaprolactone nanofibers incorporated with aster yomena extract and found values of 2.5 and 3.7 MPa. Tavares et al.^71^ evaluated the elongation at break of chitosan-zein films with different concentrations of ellagic acid nanoparticles and reported values of 18.52 ± 4.9 and 5.16 ± 1.83%, with a significant reduction by the increase of ellagic acid concentration.^5^ The improvement in tensile strength was related to the composition of the extract, which provided intermolecular hydrogen bonding between the film-forming substances and hindered the free rotation of bonds in films.

The carboxylic groups characteristic of anthocyanins, identified in the active membrane by FTIR analysis, are able to interact with the biopolymeric matrix through hydrogen bonding, leading to a more compact and organized polymer network and, consequently, increased tensile strength.^49^ This fact is supported by the observed peak shifts in the FTIR spectra of the active membrane compared with the lyophilized extract, indicating intermolecular interactions. Regarding elongation at break, no significant differences were observed between the membranes with and without the extract. Although some components of natural extracts have been reported to exert a plasticizing effect,^72^ the unchanged elongation at break in this system can be attributed to a balance between noncovalent reinforcing interactions, which increase tensile strength, and plasticizing effects, in which phenolic molecules occupy free space within the polymer matrix and share noncovalent bonds. As highlighted by Nastasi et al.,^73^ the influence of polyphenols on the plasticizing behavior of biopolymeric matrices is highly complex and strongly dependent on their concentration and chemical diversity^74^.

The top-down morphology of the bilayer membranes was investigated to evaluate the zein fibers formed (second layer) on the chitosan film (first layer). The corresponding micrographs are presented in Figure 3.

**Figure 3:**
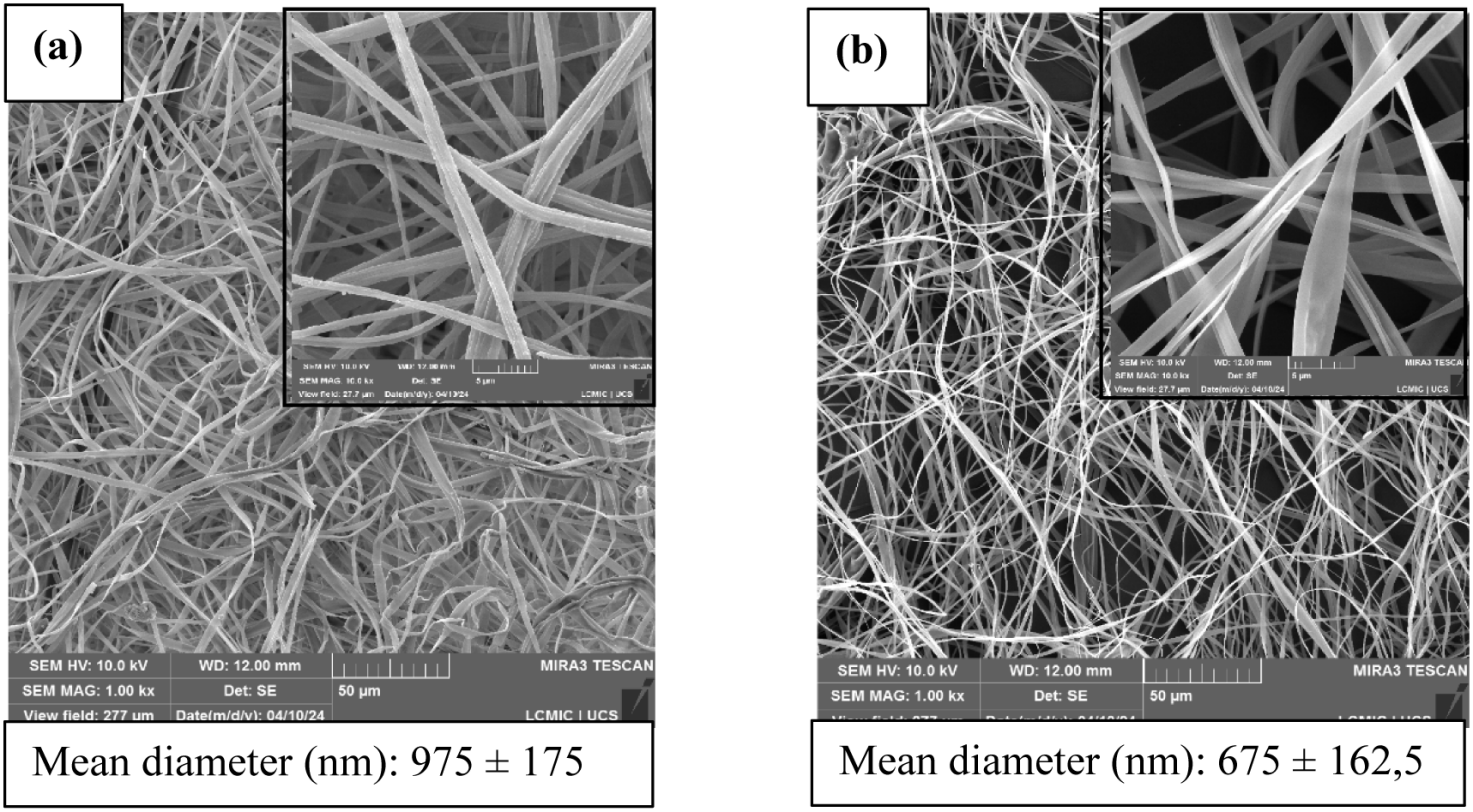
Morphology of CBM (a) and ABM (b).

In the SEM micrographs, it is evident that both fibers present a continuous and homogeneous morphology without the presence of beads. Furthermore, it is possible to infer that the incorporation of LJEE into the biopolymer matrix did not cause structural defects or visible changes in the overall organization of the fibers. Beyond the mean values, the diameter distribution analysis revealed a narrower dispersion for LJEE-containing fibers, indicating that the extract promoted a more uniform electrospinning process.

This phenomenon is likely related to the chemical composition of LJEE, whose phenolic components may interact with the biopolymer matrix and influence key solution properties during electrospinning. Previous studies^10–19^ also observed a diameter reduction in zein fibers loaded with jaboticaba extracts and attributed this behavior to increased apparent viscosity and electrical conductivity caused by the incorporation of phenolic compounds. A narrower distribution and decreased diameter typically indicate improved jet stability, leading to more consistent fiber stretching and solidification.

In comparison with the broader literature,^49^ developed zein/polyoxyethylene core–sheath ultrathin fibers and found diameters ranging from 117 ± 22 nm to 2460 ± 447 nm. Additionally, Moeini et al.^72^ produced electrospun zein fibers exposed to glutaraldehyde for enzyme immobilization and reported values between 380 and 900 nm. Therefore, the diameter values obtained in the present work are consistent with previous reports while remaining on the lower end of the typical range. Beyond reinforcing the structural quality of the fibers, the reduced and more uniform diameter profile also suggests promising potential for application in the controlled delivery of active compounds entrapped within the nanofibers.

The functional groups of the samples were evaluated using FTIR analysis and compared with the spectra of LJEE, chitosan film (first layer), and chitosan and zein powder. The FTIR spectra are listed in Figure 4.

**Figure 4:**
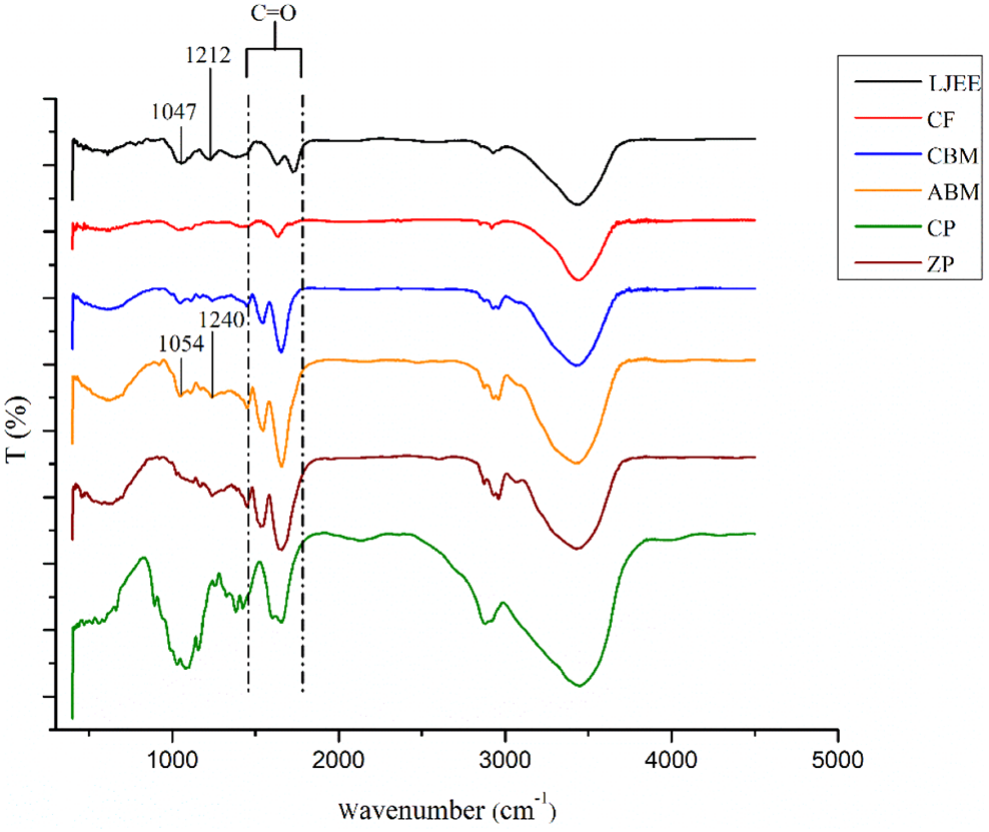
FTIR spectra of lyophilized jaboticaba epicarp extract (LJEE), chitosan film (CF), control bilayer membrane (CBM), active bilayer membrane (ABM), chitosan powder (CP), and zein powder (ZP).

It is possible to observe bands around 1540 and 1661 cm^–1^ for all biopolymeric samples. This fact can be attributed to the characteristics of chitosan and zein biopolymers, such as the C=O stretching vibration of amide I and the N–H bending and C–N stretching vibration of amide II.^66^ Regarding the LJEE, these bands appear at 1624 and 1730 cm^–1^ and can be attributed to the C=O group of the carboxylic acid, which is also present in the anthocyanin structure and gallic acid, compounds identified in the HPLC analysis. Similar results were reported by Avila et al.^20^ Other important vibrations can be observed in the LJEE spectra, around 1047 and 1212 cm^–1^, that, according to da Rosa et al.^75^ can be associated with the elongation of the C–O–C bond in anthocyanins. These peaks can also be observed in the ABM spectra; however, in this case, the incorporation of LJEE into the biopolymeric matrix caused a shift to 1054 and 1240 cm^–1^. This behavior indicates a probable interaction between compounds of LJEE and biopolymer. Finally, the peaks in the region of 3398–3437 cm^–1^ could be attributed to the lengthening of O–H in carboxylic groups.^76,77^

The bilayer membranes, as well as the chitosan film, were analyzed for their thermal stability, and the results are shown in Figure 5 through the TG and DTA curves.

**Figure 5:**
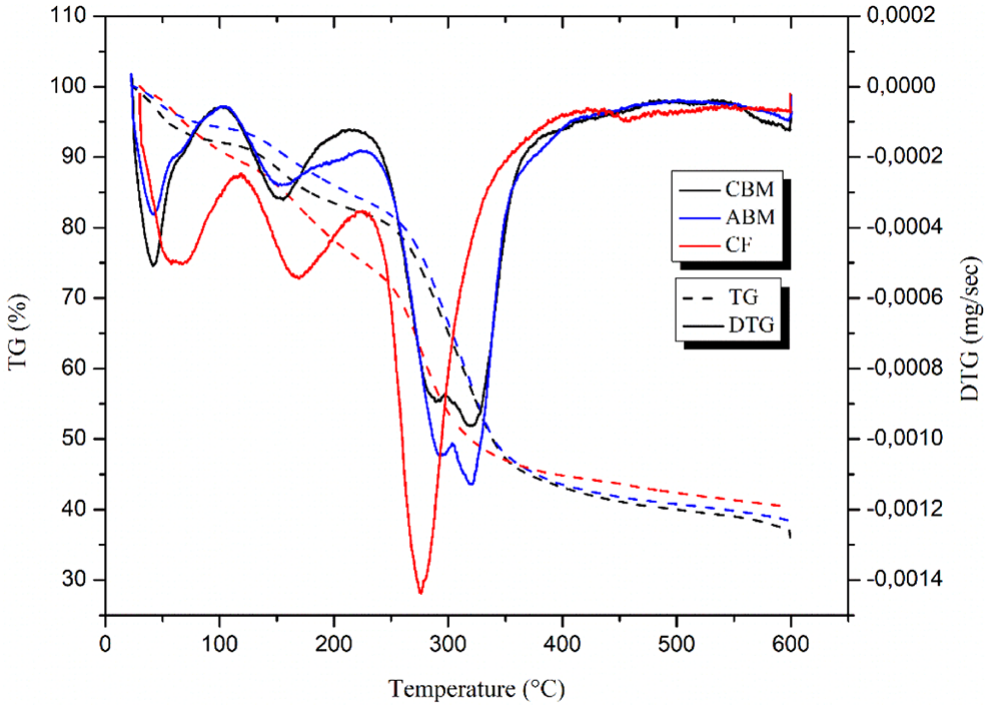
Thermogravimetric curves of control bilayer membrane (CBM), active bilayer membrane (ABM), and chitosan film (CF).

TGA and DTA measurements (Figure 5) indicate similar curve profiles for ABM and CBM within the measured temperature (up to 600 °C) with multistage thermal decomposition. All samples started losing weight before 100 °C, with the weight loss rate increasing in this temperature range, which is mainly due to water evaporation.^8,65^ The second weight loss, between 150 and 170 °C, is noted, which represents the thermal decomposition of glycerol, a constituent in chitosan films.^17,78^ Finally, the last and most pronounced weight loss appears around 250 °C for chitosan film and 260 to 320 °C for bilayer membranes, which is due to the decomposition of the biopolymers, both chitosan and zein. In this decomposition range, high-temperature-induced acetylation of chitosan and complex decomposition of zein occur.^8,65,70^

Both CBM and ABM showed an increase in thermal stability compared to CF, evidenced by changes in the initial decomposition temperatures of biopolymers (250 °C for the CF sample and 260 to 320 °C for the CBM and ABM samples), as evidenced by changes in the initial decomposition temperature of the samples. This result suggested good compatibility between the biopolymers that make up the bilayer structure, promoting improvements in the thermal properties of the materials. Similar to these results reported by Neto et al.,^79^ who evaluated chitosan and konjac glucomannan, as well as the blend film of these biopolymers.

Thus, the incorporation of LJEE did not significantly affect the thermal stability of the bilayer membranes, since the decomposition temperature remained the same, around 280 °C. Similar behavior was reported by de Souza Falcão et al.,^80^ who attributed this fact to the low concentration of bioactive compounds compared to the other components of biopolymeric materials.

The bilayer membranes were also evaluated for the migration of bioactive compounds in different food simulants, and the results are shown in Table 3.

**Table 3:** Active Potential of ABM According to Migration Test[Table-fn tbl3fn1]

	Acetic acid 3% (v/v)	Oil	Water	Etanolic solution 10% (v/v)
AA (%)	13.22 ± 0.56^a^	41.73 ± 0.21^b^	13.69 ± 0.03^a^	12.06 ± 2.14^a^
TPC (mg_GAE_ g^–1^)	1.91 ± 0.12^a^	10.32 ± 1.17^b^	4.82 ± 0.06^a^	4.30 ± 0.53^a^

i Mean ± standard deviation (*n* = 3). Different letters in the same line indicate significant differences between the samples for the Tukey test (*p* < 0.05).

According to Table 3, a greater release was observed when the ABM was placed in contact with the oil than with the other food simulants. This fact may be related to the interaction between oleic acid and the structure of zein, the biopolymer that constitutes the layer containing the extract. Wang et al.^81^ studied the effect of hydrophilic and lipophilic compounds on zein microstructures and reported that the presence of oleic acid caused some effects on the zein structure, which was initially coated by oleic acid, fused, and then transformed into a sponge-like structure.

Furthermore, the result for antioxidant activity is similar to that reported by Avila et al.,^6^ who reported 41.84 ± 1.59% for the carrageenan films incorporated with jaboticaba epicarp extract. Regarding total phenolic content, Rodrigues et al.^82^ developed chitosan-gelatin membranes with different concentrations of jaboticaba epicarp extract and reported values ranging from 0.55 ± 0.04 to 1.79 ± 0.07 mg_GAE_ g^–1^.

On the basis of this, the oil was chosen as the food simulant to be used in the microbiological analysis. This analysis was carried out against *S. aureus* and *E. coli*, and the results are presented in Table 4.

**Table 4:** Microbial Inhibition (%) of Food Simulant (Oil) and Bilayer Membranes Against *S. Aureus* and *E. Coli* [Table-fn tbl4fn1]

	*S. aureus*	*E.coli*
Pure Oil	54.37 ± 0.09^a^	20.23 ± 1.44^a^
CBM	59.97 ± 1.01^b^	26.38 ± 0.84^a^
ABM	71.86 ± 2.06^c^	46.23 ± 3.17^b^

i Mean ± standard deviation (*n* = 3). Different letters in the same column indicate significant differences between the samples for the Tukey test (*p* < 0.05).

The results shown in Table 4 indicate significant differences for all samples against *S. aureus*. Although the oil simulant exhibits intrinsic antimicrobial activity against *S. aureus* and *E. coli*, as previously reported by Rodrigues et al.,^82^ the inhibition observed for the CBM sample was significantly greater than that obtained for pure oil, which may be attributed to the antimicrobial properties of chitosan.^14,15^ Chitosan contains protonated amino groups under acidic conditions that can interact electrostatically with the negatively charged bacterial cell surfaces, leading to membrane destabilization, impaired nutrient transport, and eventual cell death.^83^

However, the most pronounced antimicrobial effect was achieved for the ABM, indicating a clear synergistic action among chitosan, the bilayer structure, and the migrated phenolic compounds. Phenolic constituents of LJEE, including gallic acid, caffeic acid, *p*-coumaric acid, and cyanidin-3-glucoside, are known to exert antimicrobial activity through multiple mechanisms.^83^ For *S. aureus*­(Gram-positive), these molecules may disrupt the thick peptidoglycan layer and increase membrane permeability, resulting in the leakage of intracellular components. In contrast, activity against *E. coli* (Gram-negative), which possesses an outer membrane that restricts molecular diffusion, may occur primarily through interference with membrane proteins essential for ion balance and metabolic function rather than direct membrane rupture.^84,85^

The behavior observed in *E. coli* reinforces this interpretation: while pure oil and CBM showed similar inhibition profiles, only ABM produced a statistically higher reduction in microbial growth, demonstrating the contribution of LJEE migration. The greater antimicrobial activity observed in the oil simulant likely reflects the higher solubility and diffusivity of hydrophobic phenolic compounds in nonpolar media, increasing their availability to interact with bacterial cells rather than an antimicrobial effect inherent to the oil itself.^83^

Previous studies support these findings, including the antimicrobial activity of jaboticaba peel extract against *E. coli* and *S. aureus* and the use of jaboticaba-derived compounds in bilayer systems.^10,19^ Notably, the ABM presented superior inhibition against *E. coli* compared to the 22% reported by Avila et al.,^10^ reinforcing the benefit of the bilayer structure as a delivery platform. Overall, the antimicrobial activity, combined with the migration profile of bioactive compounds, quantified through antioxidant activity and total phenolic content, supports the suitability of ABM as an active packaging material. Considering that oil is a recognized simulant of fatty foods according to RDC no. 51,^33^ the developed material shows potential to minimize spoilage associated with microbial proliferation and lipid oxidation.

## 4 Conclusions

In this work, the potential use of LJEE as a natural additive in the formulation of materials for food packaging was investigated. Furthermore, the phenolic profile demonstrated the presence of important bioactive compounds, such as gallic acid, caffeic acid, *p*-coumaric acid, trans-ferulic acid, kaempferol, and cyanidin-3-glucoside. Considering the potential for applying the extract as a natural additive, we developed bilayer membranes. Surface tension and contact angle tests were applied, and it was found that the addition of the extract significantly affected the surface tension of the biopolymeric solutions. The results indicate a tendency toward the formation of fibers without granules. On the other hand, it was also possible to observe a significant change in the contact angle by the addition of the extract in the zein solution. The good adhesion between the layers (zein fiber and chitosan film) was confirmed by the hydrophilic character of the chitosan film in the presence of both zein solutions. The characterization of the bilayer membranes indicates a reduction, due to the addition of the extract, in important parameters for the application for which the material is intended. These parameters being solubility in water and water-vapor permeability. The tensile strength was significantly increased by the addition of the extract, while the elongation at break was not significantly influenced. Regarding morphology, it was possible to observe the obtaining of homogeneous fibers without beads, corroborating the results of the surface tension of the polymeric solutions. Through FTIR analysis, it is possible to identify functional groups characteristic of biopolymers and the extract, as well as confirm the presence of the extract in ABM. The thermal stability of bilayer membranes was higher than that of the chitosan film, indicating good compatibility of the biopolymers, while the addition of the extract did not affect this property of the bilayer membrane. The migration of bioactive compounds present in ABM showed that the best migration was obtained in oil, with TPC and AA values being higher than those observed in the other food simulants. The antimicrobial activity of ABM when in contact with oil was carried out, and the results showed important inhibition against the microorganisms *S. aureus* and *E. coli*. Thus, the potential application of jaboticaba epicarp extract as a natural additive in the development of packaging materials is notable, proven by the active potential of the bilayer membrane, both due to its antioxidant and antimicrobial actions. Therefore, the material developed showed promising characteristics for application as active food packaging, especially intended for fatty foods, and could minimize losses due to oxidation and microbial contamination.
